# Thinking on Transcranial Direct Current Stimulation (tDCS) in Reading Interventions: Recommendations for Future Research Directions

**DOI:** 10.3389/fnhum.2019.00157

**Published:** 2019-05-22

**Authors:** Yongjun Zhang, Hongwen Song, Ying Chen, Lin Zuo, Xinzhao Xia, Xiaochu Zhang

**Affiliations:** ^1^School of Foreign Languages, Anhui Jianzhu University, Hefei, China; ^2^Centers for Biomedical Engineering, School of Information Science and Technology, University of Science and Technology of China, Hefei, China; ^3^School of Humanities and Social Science, University of Science and Technology of China, Hefei, China; ^4^CAS Key Laboratory of Brain Function and Disease, School of Life Science, University of Science and Technology of China, Hefei, China; ^5^Hefei Medical Research Center on Alcohol Addiction, Anhui Mental Health Center, Hefei, China; ^6^Academy of Psychology and Behavior, Tianjin Normal University, Tianjin, China; ^7^Hefei National Laboratory for Physical Sciences at the Microscale and School of Life Sciences, University of Science and Technology of China, Hefei, China

**Keywords:** reading difficulties, tDCS, reading interventions, opinion, future directions

## Introduction

Transcranial direct current stimulation (tDCS) is a non-invasive neural modulation technique to remediate many neural deficits. tDCS involves the application of a direct current (1–2 mA) which modulates the resting membrane potential of cortical neurons (Dasilva et al., 2011; Brunoni et al., 2012). In tDCS, anodal stimulation results in subthreshold depolarization and increases the likelihood of neurons' firing, while cathodal stimulation hyperpolarizes neurons and decreases the likelihood of their firing (Nitsche et al., 2003; Stagg and Nitsche, 2011). The outlasting neuroplastic effects of tDCS depend on synaptic plasticity of glutamatergic neurons (Liebetanz et al., 2002; Nitsche et al., 2003). Furthermore, magnetic resonance spectroscopy (MRS) studies have shown reduction in gamma-aminobutyric acid (GABA) and increase in glutamate following tDCS (Rae et al., 2009; Stagg et al., 2009), and tDCS can modulate postsynaptic connectivity within brain networks (Stagg and Nitsche, 2011; Meinzer et al., 2014). Therefore, it is thought that the long-term effects of tDCS may share common characteristics with long-term depression (LTD) and long-term potentiation (LTP) in the regulation of neuroplasticity (Elmasry et al., 2015).

Functional imaging studies have converged in identifying a left-hemisphere lateralized reading network, including the inferior frontal gyrus, the occipito-temporal region, and the parieto-temporal region (Gabrieli, 2009; Richlan et al., 2010, 2011; Martin et al., 2015). Atypical activations of these regions in reading tasks have been reported in individuals having reading difficulties (Horwitz et al., 1998; Paulesu et al., 2001; Mccandliss and Noble, 2003; Hoeft et al., 2007; Morken et al., 2017).

In view of the neural modulation property of tDCS and the benefits of applying tDCS to modulating reading efficiency in healthy subjects (Antal et al., 2014; Thomson et al., 2015), several studies have adopted tDCS in reading interventions among the individuals with developmental dyslexia (DD) and below-average readers (Turkeltaub et al., 2012; Heth and Lavidor, 2015; Costanzo et al., 2016a,b, 2018; Younger et al., 2016). These studies mainly follow the traditional assumption that anodal/cathodal stimulation increases/decreases cortical neural excitability, and in these studies, the anodal/cathodal electrodes (5 × 5 cm or 5 × 7 cm) were placed over the targeted areas generally set within the temporo-parietal cortex. However, the observed enhancement of specific reading abilities (mainly including grapheme-to-phoneme mapping, phonological processing, reading efficiency, rhyming judgment) was inconsistent across studies, and in some cases the effect sizes of the same outcome measure were quite different between studies (Turkeltaub et al., 2012; Younger et al., 2016). Furthermore, due to the distributed feature of traditional tDCS and the fact that no neural evidence was reported in the results of existing reading-remediation studies, we cannot rule out the possibility that the outcomes may also in part arise from the stimulation of regions adjacent to the target area. Therefore, we herein put forth our opinion on issues concerning stimulation parameters, populations, defocusing, combination of tDCS with cognitive training, and outcome measures, which may influence the evaluation of tDCS-based reading interventions and should be considered more carefully in future studies. Both dyslexics and readers below the average reading level are categorized as individuals with reading difficulties in this paper.

## Stimulation Parameters

The variable and inconsistent findings of tDCS-based reading interventions may relate closely to some stimulation parameters, including current intensity, number of sessions, and session duration. Current intensities of 1 mA (Costanzo et al., 2016a,b, 2018) or 1.5 mA (Turkeltaub et al., 2012; Heth and Lavidor, 2015; Younger et al., 2016) were used in available studies, and current density varied between 0.04 and 0.06 mA/cm^2^. Although higher current density may yield larger cognitive effects (Boggio et al., 2006; Teo et al., 2011), it should be adopted with caution in that higher density may cause skin burns (Palm et al., 2008), interfere with double blinding (O'Connell et al., 2012), and influence activities in regions deeper than those to be targeted (De Aguiar et al., 2015). We suggest that future tDCS-based reading interventions use more randomized controlled trials (RCTs) to obtain an optimal balance between stimulation intensity and intervention effect.

Number of sessions is another critical issue that may influence tDCS effect. Two review studies found no evidence of a reliable effect of single-session tDCS on cognitive performance in healthy participants (Horvath et al., 2015; Westwood and Romani, 2017), and another systematic review suggested that tDCS comprising multiple sessions can ameliorate symptoms of several psychiatric disorders (Kekic et al., 2016). Therefore, it is also worth our investigation to figure out a more feasible number of tDCS sessions among people with reading difficulties. However, the variable results of single session (Turkeltaub et al., 2012; Costanzo et al., 2016b; Younger et al., 2016) or multiple-session tDCS-based reading interventions (Heth and Lavidor, 2015; Costanzo et al., 2016a, 2018) were not expounded from the perspective of this issue. In view of the limited studies within the scope under discussion, more studies using between-subject design or just replicating prior work are required to draw a general conclusion on the issue of session number.

The studies under discussion didn't vary too much regarding the parameter of session duration, and 20 min was a common adoption. Given that relatively longer durations (above 10 min) determine larger effects (Hill et al., 2016; Giulia et al., 2019), 20 min duration can be still feasible in future reading interventions. Duration between 10 and 20 min, for example, 15 min, may be preferable if the effect is not inferior to that with 20 min. As for those studies consisting of several stimulation sessions (Heth and Lavidor, 2015; Costanzo et al., 2016b, 2018), interval between sessions (IBS) is also an important factor worth consideration. Research has shown that the IBS may influence tDCS outcome (Monte-Silva et al., 2010), and the inhibitory effect of cathodal-tDCS was delayed while the excitatory effect of anodal-tDCS converted into inhibition with a 24-h IBS (Monte-Silva et al., 2013). Based on available findings, it is suggested that multiple sessions with a daily frequency and a 1-week washout period seem to be suitable for populations with language disorder (De Aguiar et al., 2015). Future tDCS-based reading interventions need to compare the effects of tDCS with different IBS, especially for those studies using crossover design in which carry-over effect is more easily to arise.

## Features of Different Groups of Populations

tDCS effects may vary between healthy participants and individuals with cognitive or neuropsychiatric disorders (Ferrucci et al., 2009; Brunoni and Vanderhasselt, 2014; Dedoncker et al., 2016). Furthermore, in two studies exploring tDCS modulatory effect on participants' reading performance, both Thomson et al. (2015) and Westwood et al. (2017) suggested that tDCS are more likely to induce effect in brains with more dysfunctional neuronal excitability, and there is less space for further improvement in already optimized brain areas. Existing tDCS-based reading interventions among dyslexics or below-average readers are more or less effective, but few of them take this issue into account in the interpretation of their results. It is possible that the lesion state or severity level of the reading-related areas is a continuum, thus future research can examine whether different stimulation dose should be applied to modulate reading efficiency in participants ranging from below-average readers to dyslexics. Notably, behavioral training may be a more feasible alternative intervention for those low-to-average readers, given that tDCS may disrupt inter-hemispheric inhibition in low-to-average readers and induced a negative effect on their phonological working memory abilities, as is shown by Younger et al. (2016).

Besides, the atypical brain areas of dyslexic populations may also vary with their different cultures and ages. Chinese dyslexic individuals exhibit atypical activation in the left prefrontal cortex (Siok et al., 2004), while French, Italian, and English readers with dyslexia exhibit hypoactivation in the left temporal cortex (Paulesu et al., 2001). Although there is a dysfunction of a left ventral occipito-temporal region in both children and adults, underactivation in bilateral inferior parietal regions was only found in children and underactivation in superior temporal cortex was only found in adults (Richlan et al., 2011). Therefore, future tDCS-based reading interventions are supposed to set different target areas and use different electrode montages in subjects from different cultures and age groups.

## Defocusing

The reduction of spatial focality and modulation efficiency at the targeted areas during tDCS is defined as defocusing, which is one notable limitation of the traditional tDCS protocol using 10–20 EEG notation (Datta et al., 2008; Boggio et al., 2009; Thomson et al., 2015). The low conductivity of skull, the electric current concentration on the edge of gyri, and the widespread excitatory effect of the anodal tDCS (Datta et al., 2009; Thomson et al., 2015) are believed to be the leading factors resulting in defocusing. Computational head models have been developed to predict a more focal directional effect of current flow under the electrodes (Wagner et al., 2007; Datta et al., 2009; Sadleir et al., 2010; Turkeltaub et al., 2012). A computational head model is a structural magnetic resonance imaging/MRI-derived finite element model of an adult's head with a high resolution (1 mm^3^), and the head is segmented into different compartments representing brain tissues (Datta et al., 2009). The finite element mesh generated from the segmented data can be exported to COMSOL Multiphysics, a finite element software package, for the computation of electric fields and the simulation of electrode montage (Datta et al., 2009; Bai et al., 2014). Computational models have been used to develop electrode configurations, and a 4 × 1 ring configuration has been shown to enhance focality by positioning a small central electrode surrounded by four return electrodes, in contrast to conventional tDCS using two large rectangular pads (Datta et al., 2009; Kuo et al., 2013; Gbadeyan et al., 2016). This ring configuration is defined as high-definition tDCS (HD-tDCS)and has been demonstrated to restrict upwards of 30% of the stimulation peak within the ring perimeter (Edwards et al., 2013).

Computational models are rarely developed to guide the tDCS current flow in reading remediation. A recent study investigated the effect of tDCS on reading abilities of children and adolescents with dyslexia, but found no positive effects on text reading and high-frequency words (Costanzo et al., 2016b). It is likely that the defocusing of the traditional tDCS utilized in this study led to the negative result with regard to grapheme identification, which is also implied by Costanzo and collaborators. Another study selected the left inferior parietal lobe (IPL) as the targeted area and measured low-to-average readers' improvement on both rhyming judgment and single word reading efficiency (Younger et al., 2016). Results showed that left IPL stimulation relatively “impairs” participants' performance on the rhyming judgment task, even though it resulted in gains in the other task. In fact, it is the left IPL that underlies the storage of short-term phonological information and verbal working memory (Ravizza et al., 2002, 2004; Yue et al., 2018) needed for the rhyming judgment task, so we propose that the unexpected effect on the rhyming task could also be partially due to the defocusing in the experiment. Consequently, similar to the study conducted by Costanzo and collaborators, the current may diffuse to adjacent parietal areas so that the unexpected result emerged. In contrast, below-average readers demonstrated improved word reading efficiency as expected by Turkeltaub et al. (2012). The authors attributed this result to a finite head model generated in the study for the computation of electric fields, which predicted a focal effect under the electrodes centering over left posterior temporal cortex (pTC) and right pTC. But the above-mentioned HD-tDCS was not adopted, which might partly explain why there was no tDCS effect on nonword reading efficiency in this study.

To date, only a few studies made a direct comparison between the effect of conventional tDCS and that of HD-tDCS, and most of these studies concluded that the effects of HD-tDCS are at least comparable to that of conventional tDCS. Notably, plastic changes in the primary motor cortex showed a more delayed peak at 30 min and longer lasting after-effects after HD-tDCS, as compared to conventional tDCS (Kuo et al., 2013). Besides, in a study aiming at remediating aphasia, change in accuracy of trained items was found to be numerically higher (although not statistically significant) for HD-tDCS compared to conventional tDCS for most patients (Richardson et al., 2015). In the light of these positive findings, HD-tDCS has its potential to be realized in future reading interventions and achieve greater gains for both dyslexics and below-average readers.

## Combining tDCS with Reading-related Cognitive Training (CT)

When tDCS sessions are given in combination with CT or other rehabilitation protocols for improving motor or cognitive performance, better outcomes are achieved than with the CT or standard rehabilitation alone (Lindenberg et al., 2010; Fridriksson et al., 2011; Ditye et al., 2012; Martin et al., 2013; Penolazzi et al., 2015; Lawrence et al., 2018). The principle underlying such combination protocol is that transcranial electric stimulation (tES) can enhance the synaptic transmission and strength in neural pathways activated by the CT, so that the endogenous activation (CT) and exogenous neuromodulation (tES) can work together to facilitate the activation of neural networks subserving cognitive functions (Miniussi and Vallar, 2011; Elmasry et al., 2015). As such, we think that the efficacy of reading interventions may also be enhanced by combining tDCS with reading-related CT or tasks.

Since the CT employed in the intervention obviously cannot encompass all the different reading tasks subjects may be faced with in everyday life, it is worth further research whether the effects of combining tDCS with CT can be transferred to non-trained tasks. Given the essential role of working memory (WM) in reading ability (Hoeft et al., 2007; Pham and Hasson, 2014), several studies investigating the effects of combining tDCS with CT on the WM and transfer effects on non-trained WM tasks may shed light on this issue. The active tDCS + CT group did show significantly greater gains than the tDCS-only group or the CT-only group in non-trained WM tasks (Martin et al., 2013; Richmond et al., 2014), but no significant outcome differences were found between the active tDCS + CT group and the sham tDCS + CT group. While another study demonstrated that in a non-trained WM task, the active tDCS + CT group performed significantly better compared to the sham tDCS + CT group (Park et al., 2013). This result may arise from the electrode montage that anodes were attached to bilateral prefrontal cortex stimulated by two stimulators and cathodes were attached to the non-dominant arm. Such a montage corresponded with the notion that the left dorsolateral prefrontal cortex (dlPFC) is responsible for verbal WM while the right dlPFC subserves spatial WM (Ruf et al., 2017). Both verbal and spatial memory are correlated with reading comprehension (Swanson and Howell, 2001; Pham and Hasson, 2014), and thus the dose of treatment were doubled.

Consequently, a combination treatment protocol can be feasible in treating reading difficulties, and unilateral tDCS combined with CT can render a transfer effect; notably, bilateral tDCS is more preferable if WM-related trainings are administered. These propositions warrant future systematic exploration. Other factors such as the timing of tDCS relative to CT, frequency of tDCS + CT sessions, the type of population targeted (below-average readers or individuals with DD) may also influence the combination treatment outcome (Elmasry et al., 2015; Cancer and Antonietti, 2018).

## Adding Outcome Measures by Using Neuroimaging Methods

Adopting behavioral remediation or instructional treatment for reading difficulties have been documented as being able to improve brain activations (Temple et al., 2003; Simos et al., 2007; Meyler et al., 2008; Richards and Berninger, 2008). However, behavioral results are mainly reported as the outcome reading measures in the existing studies of tDCS-based reading remediation. Given that tDCS is a technique modulating neural activity, we propose that neuroimaging techniques such as electroencephalography (EEG), fMRI, functional near infrared spectroscopy (fNIRS) should be used to explore the neural activity changes after tDCS remediation.

Evidence has shown EEG + tDCS, fMRI+ tDCS, or fNIRS + tDCS can be used to monitor tDCS-induced changes of the neural activities involved in sustained attention (Miller et al., 2015), semantic processing (D'Mello et al., 2017), and spatial working memory (McKendrick et al., 2015), all of which have also been found to be impaired in the individuals with reading difficulties (Schulz et al., 2008; Pham and Hasson, 2014; Staels and Van den Broeck, 2017). As such, those neuroimaging methods can also be integrated into future tDCS-based reading interventions to detect the potential neural changes, which can be taken as an additional outcome measure. Apart from the above-mentioned potential neural changes, future studies can try to investigate whether other reported atypical cortical band activations (Sklar et al., 1972; Fein et al., 1986; Spironelli et al., 2008; Penolazzi et al., 2010; Papagiannopoulou and Lagopoulos, 2016), event-related potentials (ERPs) (Horowitz-Kraus, 2016), and brain activations (for a review, see Gabrieli, 2009) in populations with reading difficulties can be normalized after tDCS-based interventions.

## Conclusions

Currently, the field of tDCS-based reading interventions is still at its infancy and the related literature is relatively sparse compared to other tDCS-based research. It is therefore too early to conclude that tDCS is generally effective in this field, and we should have an objective assessment of the limitations of this technique and of the conditions in which there is limited or even negative effect. In this opinion article, we have put forth some recommendations that merit more attention in future tDCS-based reading interventions. Further evidence from larger-scale RCTs is especially required for those dyslexic individuals so as to define reproducible stimulation parameters for certain groups of samples. Based on empirical evidence and safe ethical grounds (Kekic et al., 2016), tDCS is expected to be a promising device in reading interventions, especially when it is given in combination with CT and neuroimaging methods.

## Author Contributions

YZ wrote the article. XZ revised each draft of the article. YZ, XZ, and HS helped to conceive the opinion. YC, LZ, XX and XZ contributed to data collection.

### Conflict of Interest Statement

The authors declare that the research was conducted in the absence of any commercial or financial relationships that could be construed as a potential conflict of interest.
