# Corrigendum: Thinking on Transcranial Direct Current Stimulation (tDCS) in Reading Interventions: Recommendations for Future Research Directions

**DOI:** 10.3389/fnhum.2021.814964

**Published:** 2022-01-07

**Authors:** Yongjun Zhang, Hongwen Song, Ying Chen, Lin Zuo, Xinzhao Xia, Xiaochu Zhang

**Affiliations:** ^1^School of Foreign Languages, Anhui Jianzhu University, Hefei, China; ^2^Centers for Biomedical Engineering, School of Information Science and Technology, University of Science and Technology of China, Hefei, China; ^3^School of Humanities and Social Science, University of Science and Technology of China, Hefei, China; ^4^CAS Key Laboratory of Brain Function and Disease, School of Life Science, University of Science and Technology of China, Hefei, China; ^5^Hefei Medical Research Center on Alcohol Addiction, Anhui Mental Health Center, Hefei, China; ^6^Academy of Psychology and Behavior, Tianjin Normal University, Tianjin, China; ^7^Hefei National Laboratory for Physical Sciences at the Microscale and School of Life Sciences, University of Science and Technology of China, Hefei, China

**Keywords:** reading difficulties, tDCS, reading interventions, opinion, future directions

There is an error in the Funding statement. The correct number for the Humanities and Social Science Research Foundation of Education Department of Anhui Province is “SK2019A0661, SK2018A0571, and XJ2018002002.”

The authors apologize for this error and state that this does not change the scientific conclusions of the article in any way. The original article has been updated.

## Publisher's Note

All claims expressed in this article are solely those of the authors and do not necessarily represent those of their affiliated organizations, or those of the publisher, the editors and the reviewers. Any product that may be evaluated in this article, or claim that may be made by its manufacturer, is not guaranteed or endorsed by the publisher.

